# Factors associated with do-not-resuscitate document completion among patients hospitalized in geriatric ward

**DOI:** 10.1186/s12877-021-02407-3

**Published:** 2021-08-25

**Authors:** Chien-Yi Wu, Chun-Hao Jen, Yun-Shiuan Chuang, Tzu-Jung Fang, Yu-Hsuan Wu, Ming-Tsang Wu

**Affiliations:** 1 Department of Family Medicine, Kaohsiung Medical University Hospital, Kaohsiung Medical University, No.100, Tzyou 1st Road, Kaohsiung, 807 Taiwan; 2 Department of Public Health, College of Health Sciences, Kaohsiung Medical University, Kaohsiung City, Taiwan; 3 Research Center for Environmental Medicine, Kaohsiung Medical University, Kaohsiung City, Taiwan; 4 Division of Geriatrics and Gerontology, Department of Internal Medicine, Kaohsiung Medical University Hospital, Kaohsiung City, Taiwan; 5 Program of Environmental and Occupational Medicine and Graduate Institute of Clinical Medicine, Kaohsiung Medical University, Kaohsiung City, Taiwan

**Keywords:** Geriatric care, Older people, Do-not-resuscitate, Advance care planning

## Abstract

**Background:**

With a rapidly aging population, there is an increasing need for do-not-resuscitate (DNR) and advance care planning (ACP) discussions. This study investigated the factors associated with signing DNR documents of older patients in the geriatric ward.

**Methods:**

We conducted a retrospective cohort study at a geriatric ward in a tertiary hospital in Southern Taiwan. Three hundred and thirty-seven hospitalized older patients aged ≥65 years in the geriatric ward from 2018 to 2019. The Hospital Information System and electronic medical records were accessed to obtain details regarding patients’ demographics, daily living activities, serum albumin level, nutrition screening score, intensive care unit transferal, resuscitation procedure, days of hospital stay, and survival status on discharge, and DNR status was recorded retrospectively. Patients were classified into DNR and non-DNR groups, with t-tests and Chi-square tests applied to compare the differences between groups. Logistic regression was performed to predict factors related to the DNR documents.

**Results:**

A total of 337 patients were included, 66 of whom had signed a DNR during hospitalization. After multivariate logistic regression, age 85 or more compared to age 65–74 (adjusted odds ratio, aOR 5.94), poor nutrition with screening score two or more (aOR 2.71), albumin level less than 3 (aOR 3.24), Charlson Comorbidity Index higher than 2 (aOR 2.46) and once transferred to ICU (aOR 5.11) were independently associated with DNR documentation during hospitalization.

**Conclusions:**

Several factors related to DNR documents for geriatric patients were identified which could provide clinical information for physicians, patients, and their families to discuss DNR and ACP.

## Background

Due to population aging and increased comorbidities, older people worldwide have more medical needs [1], with the interdisciplinary healthcare team typically using a comprehensive geriatric assessment (CGA) to provide holistic care for older patients. Geriatricians attempt to import advance care planning (ACP) with their patients and families while under certain clinical conditions, such as advanced cancer or poor functional status [2, 3]. The ACP is an important process for patients and their relatives to discuss patients’ disease trajectory and future care plan, including end-of-life care preference with their medical teams [4]. Older patients, their relatives, or medical proxy can sign do-not-resuscitate (DNR) documents to avoid patients receiving cardiopulmonary resuscitation (CPR) during illness. In a study conducted in a tertiary academic acute care hospital, the documentation of DNR orders was significantly higher for patients under a geriatrician hospitalist service compared to staff or non-staff hospitalist service (31.6% vs. 15.7 and 10.6%, *p* < 0.001) [5]. In another study conducted in a Level I trauma center, there was a significant increment in the percentage of DNR or did not intubate, from 10.23% in the pre-intervention group to 38.22% in the post-intervention group (*p* < 0.01) [6].

In a study of 1411 patients with cancer or other life-limiting diseases, or decreased activities of daily living (ADL), an ACP meeting was arranged to decide end-of-life care by signing advance directives (AD). Patients aged 85 or more accounted for 40.3% of the population and were positively related to AD completion (aOR = 1.8, 95% CI 1.21 to 2.67, *p* = 0.003) [7]. A Canadian study in primary care practice showed that an age per 10-year increase was significantly associated with the completion of AD (aOR 1.55, 95% CI 1.26 to 1.90; *p* < 0.001) [8]. Thus, it is evident that older adults are more likely to think about ACP and sign DNR or AD.

However, end-of-life choices, including a resuscitation plan and ACP, are not formally discussed in the CGA performed by most medical facilities. Indeed, it is not routinely assessed in geriatric department facilities. A study that reviewed medical records at a geriatric primary care clinic of 98 patients aged 65–94 revealed that only 34 patients (34.7%) had documentation of AD or power of attorney (POA) [9]. In an Australian study of residents in general practices, hospitals, and aged care facilities in Australia, the rate of at least one ACP was 29.8%, statutory ACP regarding a substitute decision-maker accounted for 10.9%, and care preference accounted for 2.9% of the total population [10]. According to the Supportive and Palliative Care Indicators Tool (SPICT™) designed by the University of Edinburgh, if the patients meet the criteria, medical professionals could initiate ACP and palliative care earlier. It has been validated in multiple populations, including the geriatric population [11]. With the increase of aging in Taiwan, the demand for palliative care also rises [12]. However, there is a lack of information about what factors affected the discussion of DNR signing in Taiwanese elder people. To help the geriatric team to evaluate older adults’ conditions and start a discussion with patients and their relatives about ACP for better end-of-life care, this study sought to determine the clinical factors and conditions in an older population during hospitalization related to signing DNR documents. We hypothesized that the clinical factors and conditions were different in the geriatric patients with or without DNR signing.

## Methods

### Study design and population

This study retrospectively collected data from patients admitted to the geriatric ward at a tertiary hospital, Kaohsiung Medical University Hospital (KMUH), in southern Taiwan. Geriatric care was provided by a multi-discipline team, including three physicians, eight nurses, one social worker, one dietitian, two rehabilitation therapists, one clinical pharmacist, and one case manager. There are 16 inpatient beds in the geriatric unit that provide comprehensive geriatric care. This study was approved by the Institutional Review Boards of Kaohsiung Medical University Hospital (IRB number: KMUHIRB-E(I)-20,210,027). Because this is a retrospective anonymous secondary data analysis, the need for a written consent form was waived. Strengthening the Reporting of Observational Studies in Epidemiology (STROBE) guidance was adopted for reporting [13] .

The data regarding patients discharged from 1st January 2018 to 31st December 2019 were collected from the electronic medical records (EMR), including the date of admission and discharge, age, gender, Eastern Cooperative Oncology Group (ECOG) grading, Activities of Daily Living (ADL) score by Barthel index, KMUH nutrition screening score, Hendrich II Fall Risk Model score, religion, level of education, living status, marital status. We also recorded the surrogate decision-makers (proxy) of the geriatric patients who were important to assist patients to make the decision of the DNR document.

We also recorded the patients’ clinical condition, including Charlson Comorbidity Index (CCI), DNR status, date of signing DNR documents, serum albumin levels, number of Emergency Department (ED) visits, and number of hospitalizations in the year before the date of admission, the chief complaint of admission, number of geriatric outpatient department (OPD) visits in a year before the date of admission, admission from ED, OPD or transfer from other wards, transfer into the intensive care unit (ICU) during hospitalization, the date of transfer to ICU, the date of transfer from ICU to the general ward, whether underwent CPR, the date of performing CPR, whether intubated, the duration of ventilator use, whether underwent defibrillation, the date of defibrillator use, whether received vasopressors, the period of receiving vasopressor and survival status on discharge. We chose the above patients’ baseline and clinical factors according to the SPICT™. The other clinical factors were also collected to examine see if they could influence DNR document completion.

The nutrition screening score, including seven items, was developed by dietitians of KMUH, modified from the Subjective Global Assessment (SGA) [14]. The total score 0–2 was considered low risk of malnutrition, 3–5 as moderate risk, and 6–7 as high risk. If the patient scored 4 points or more, the dietitian would provide nutrition supply advice. The patient was awarded 1 point when meeting the following conditions: body mass index (BMI) less than 18.5; body weight (BW) loss more than 3 kg in 1–3 months or BW loss of more than 4.5 kg in 4–6 months; fat loss over the face, muscle loss over biceps or triceps, or pitting edema; oral intake amount less than half of regular, feeding tube use, total parenteral nutrition (TPN) for more than 3 days or nil per os (NPO) for more than 3 days; vomiting or diarrhea for more than 3 days, abdominal flatus, constipation or dysphagia; coma or ascites present due to liver cirrhosis, chronic kidney disease (CKD) stage 5, burn injury, multiple trauma, pressure ulcer grading 3–4, cachexia, diagnosed with cancer, post organ transplant or dementia; the amount of physical activities half as usual or mostly confined in bed.

The Hendrich II Fall Risk Model consists of eight items, including confusion, disorientation or impulsivity (4 points); symptomatic depression (2 points); altered elimination (1 point); dizziness or vertigo (1 point); gender male (1 point); use of antiepileptics (2 points); use of benzodiazepines (1 point); get-up-and-go test (4 points). A high risk of falling was considered in patients with a total score of 5 or more [15].

The lowest serum albumin level was recorded between 1 month before admission and 1 month after admission. The survival status on discharge was classified as surviving, death, and critical AAD (against advice discharge). Due to the traditional Taiwanese concept and preference of dying at home instead of the hospital, we arranged critical AAD for the terminally ill patients who hoped to die at home, thus, we viewed critical AAD as death at discharge.

### Statistical analysis

Analyses were performed using SPSS 20.0® (SPSS Inc., IL, US). The Student t-test and Chi-squared test were used to compare numerical and categorical data between two groups, respectively, while a multiple logistic regression model was used for factors related to signing a DNR. A *p*-value of less than 0.05 was considered significant.

## Results

### Study subjects

**Fig. 1:**
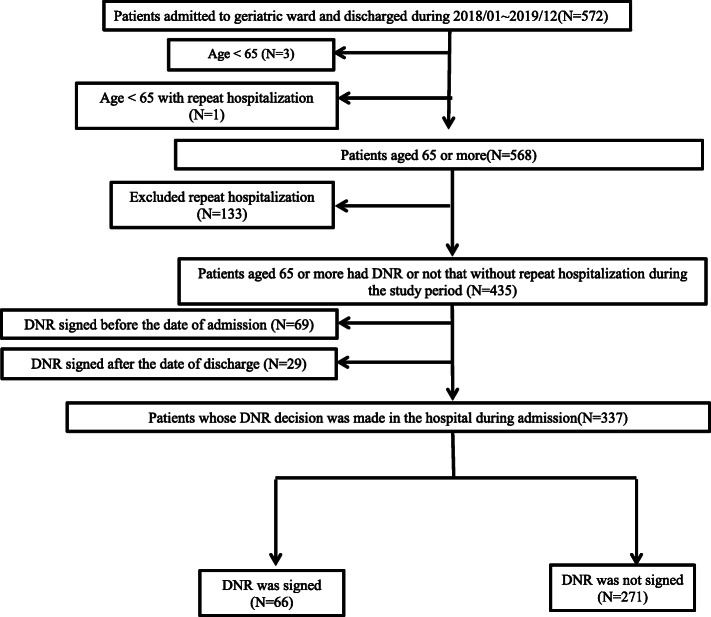
Process of case enrollment. Abbreviation: DNR, Do-Not-Resuscitate

Between January 2018 and December 2019, 572 patients were hospitalized at the geriatric wards of KMUH (Fig. 1). Those aged below 65 years old or with duplicate identity numbers (IDs) due to multiple admissions were excluded, and 435 patients were left. Sixty-nine patients whose DNR documents were signed before admission (January 2018) and 29 after discharge (December 2019) were also excluded, so a total of 337 patients were involved in this study. Patients were classified into two groups, a DNR and a non-DNR group. A total of 66 patients had their DNR documents signed during hospitalization, and there are 271 patients in the non-DNR group, meaning that the patients or their families/proxies never signed DNR documents in the hospital during the study period. The demographics and clinical variables during the DNR group’s hospitalization and the last hospitalization data of the non-DNR group were collected.

### Demographic and clinical characteristics

Tables 1 and 2 show the demographic and clinical characteristics of the enrolled patients (*n* = 337). Compared to the non-DNR group, the DNR group was significantly older (85.5 ± 7.0 vs. 80.3 ± 8.0, *p* < 0.001) with fewer admissions from the ED (60.6% vs. 74.2%, *p* = 0.008), and more from other wards (25.8% vs. 11.1%, *p* = 0.008). There were also more visits to the geriatric OPD in the year before admission in the DNR group (3.2 ± 3.8vs. 2.0 ± 3.6, *p* = 0.022). The performance status was significantly worse in the DNR group by ADL score (27.8 ± 26.5 vs. 58.6 ± 33.1, *p* < 0.001). 

The DNR group had a higher nutrition score of 4 or more and needs for dietitian consultation (30.3% vs. 15.5%, *p* = 0.005). Furthermore, the DNR group also had a higher nutrition screening score of 2 or more than the non-DNR group (75.8% vs. 45.8%, *p* < 0.001). Serum albumin was recorded in 247 patients, with the DNR group lower than the non-DNR group (2.7 ± 0.4 g/dL vs. 3.3 ± 0.5 g/dL, *p* < 0.001). The Pearson correlation to compare serum albumin level and nutrition score was − 0.22 (*p* < 0.001), indicating that the higher the nutrition screen score, the lower the albumin level. Regarding CCI, the DNR group had a higher score (3.7 ± 2.4 vs. 2.4 ± 1.8, *p* < 0.001). Also, the DNR group had a more extended hospital stay (24.3 ± 21.2 vs. 11.5 ± 9.9, *p* < 0.001), with more cases transferred to ICU during hospitalization (30.3% vs. 6.6%, *p* < 0.001) and the signing of all DNRs occurred after transfer to ICU. Furthermore, more patients in the DNR group received vasopressor treatment during hospitalization (7.6% vs. 2.2%, *p* = 0.028) and had a poorer survival status on discharge (77.3% vs. 98.9%, *p* < 0.001). 

There were no significant differences between groups regarding gender, level of education, marital status, living status, surrogate decision maker, religion, Hendrich II Fall Risk Model, and the number of visits to the ED and admission in the year before the date of admission, as well as the number of patients undergoing intubation, chest compression, and defibrillator use. Also, there was no significant difference in days of ventilator and vasopressor use.

**Table 1:** Demographic characteristics of enrolled patients (*n* = 337)

Variables	Total	DNR document		*p*-value
		Yes (*n* = 66)	No (*n* = 271)	
**Age**	81.3 ± 8.1	85.5 ± 7.0	80.3 ± 8.0	< 0.001
**Age stratification**				< 0.001
65–74	73(21.7%)	4(6.1%)	69(25.5%)	
75–84	139(41.2%)	24(36.4%)	115(42.4%)	
≥ 85	125(37.1%)	38(57.6%)	87(32.1%)	
**Gender**				0.82
Female	198(58.8%)	38(57.6%)	160(59.0%)	
Male	139(41.2%)	28(42.4%)	111(41.0%)	
**Education**				0.45
≤ 6 years	234(69.4%)	49(74.2%)	185(68.3%)	
6 ~ 12 years	77(22.8%)	14(21.2%)	63(23.2%)	
> 12 years	26(7.7%)	3(4.5%)	23(8.5%)	
**Marriage**				0.87
Unmarried	4(1.2%)	1(1.5%)	3(1.1%)	
Married	154(45.7%)	29(43.9%)	125(46.1%)	
Widowed	170(50.4%)	35(53.0%)	135(49.8%)	
Separated / divorced	9(9%)	1(1.5%)	8(3.0%)	
**Living status**				0.40
Alone	18(5.3%)	2(3.0%)	16(5.9%)	
Live with family	280(83.1%)	54(81.8%)	226(83.4%)	
Live in institution	39(11.6%)	10(15.2%)	29(10.7%)	
**Surrogate decision-maker (proxy)**				0.21
Spouse	35(10.4%)	3(4.5%)	32(11.8%)	
Child	272(80.7%)	56(84.8%)	216(79.7%)	
Others	30(8.9%)	7(10.7%)	23(8.5%)	
**Religion**				0.79
Nullifidian	49(14.5%)	10(15.2%)	39(14.4%)	
Christian	16(4.7%)	4(6.1%)	12(4.4%)	
Buddhism	106(31.5%)	19(28.8%)	87(32.1%)	
Taoism/Taiwanese folk religion	158(46.9%)	32(48.5%)	126(46.5%)	
Catholicism	4(1.2%)	0(0%)	4(1.5%)	
Others	4(1.2%)	1(1.5%)	3(1.1%)	

Data presented as mean ± standard deviation (SD) for continuous variables and frequency (percentage, %) for categorical variables. The *p*-values were calculated using the t-test for continuous variables and the Chi-squared test for categorical variables.

**Table 2:** Clinical characteristics of enrolled patients (*n* = 337)

Variables	Total	DNR document		*p*-value
		Yes (n = 66)	No (n = 271)	
**Geriatric hospitalization source**				0.008
ED	241(71.5%)	40(60.6%)	201(74.2%)	
OPD	49(14.5%)	9(13.6%)	40(14.8%)	
Transfer from other department	47(13.9%)	17(25.8%)	30(11.1%)	
**ECOG stratification**				< 0.001
0–1	174(51.6%)	21(31.8%)	153(56.5%)	
2–4	163(48.4%)	45(68.2%)	118(43.5%)	
**ADL**	52.6 ± 34.2	27.8 ± 26.5	58.6 ± 33.1	< 0.001
**ADL stratification**				< 0.001
< 50	168(49.9%)	50(75.8%)	118(43.5%)	
≥ 50	169(50.1%)	16(24.2%)	153(56.5%)	
**Nutrition Score**	1.9 ± 1.6	2.6 ± 1.5	1.77 ± 1.6	< 0.001
**Nutrition Score stratification(2)**				< 0.001
< 2	163(48.4%)	16(24.2%)	147(54.2%)	
≥ 2	174(51.6%)	50(75.8%)	124(45.8%)	
**Albumin level(g/dL)**	3.2 ± 0.5	2.7 ± 0.4	3.3 ± 0.5	< 0.001
**Albumin stratification**				< 0.001
< 3(g/dL)	92(27.3%)	41(62.1%)	51(18.8%)	
≥ 3(g/dL)	155(46.0%)	21(31.8%)	134(49.4%)	
missing	90(26.7%)	4(6.1%)	86(31.7%)	
**Fall Risk Score**	3.5 ± 2.6	3.1 ± 2.4	3.6 ± 2.6	0.241
**CCI**	2.6 ± 2.0	3.7 ± 2.4	2.4 ± 1.8	< 0.001
**CCI stratification**				< 0.001
≤ 2	182(54.0%)	21(31.8%)	161(59.4%)	
> 2	155(46.0%)	45(68.2%)	110(40.6%)	
**Hospitalization day**	14.0 ± 13.8	24.3 ± 21.2	11.5 ± 9.9	< 0.001
**Hospitalization day stratification**				< 0.001
≤ 10	182(54.0%)	15(22.7%)	167(61.6%)	
> 10	155(46.0%)	51(77.3%)	104(38.4%)	
**Geriatric OPD times in the past one year**	2.2 ± 3.7	3.2 ± 3.8	2.0 ± 3.6	0.02
**Geriatric OPD**				0.015
No	188(55.8%)	28(42.4%)	160(59.0%)	
Yes	149(44.2%)	38(57.6%)	111(41.0%)	
**ED or hospitalization times in the past one year**	2.4 ± 2.6	2.9 ± 2.8	2.3 ± 2.6	0.09
**ICU admission**				< 0.001
No	299(88.7%)	46(69.7%)	253(93.4%)	
Yes	38(11.3%)	20(30.3%)	18(6.6%)	
**ICU days**	6.3 ± 5.1	7.3 ± 5.5	5.1 ± 4.5	0.19
**Intubation**				0.07
No	313(92.9%)	58(87.9%)	255(94.1%)	
Yes	24(7.1%)	8(12.1%)	16(5.9%)	
**Days of Intubation**	4.5 ± 4.5	7.7 ± 5.8	2.8 ± 2.7	0.052
**Chest percussion**				0.48
No	335(99.4%)	66(100%)	269(99.3%)	
Yes	2(0.6%)	0(0%)	2(0.7%)	
**Defibrillation**				0.62
No	336(99.7%)	66(100%)	270(99.6%)	
Yes	1(0.3%)	0(0%)	1(0.4%)	
**Vasopressor use**				0.02
No	326(96.7%)	61(92.4%)	265(97.8%)	
Yes	11(3.3%)	5(7.6%)	6(2.2%)	
**Days of vasopressor use**	1.7 ± 2.4	2.6 ± 3.5	1.0 ± 0.0	0.37
**Survival status**				< 0.001
Survival	319(94.7%)	51(77.3%)	268(98.9%)	
Death	18(5.3%)	15(22.7%)	3(1.1%)	

Data presented as mean ± standard deviation (SD) for continuous variables and frequency (percentage, %) for categorical variables. The *p*-values were calculated using the t-test for continuous variables and the Chi-squared test for categorical variables.

*Abbreviation*: *ED* Emergency Department, *OPD* Outpatient Department, *ADL* Activity of Daily Living, *ECOG* Eastern Cooperative Oncology Group performance status, *CCI* Charlson Comorbidity Index, *ICU* Intensive Care Unit

### Factors associated with DNR documents

Table 3 shows the predictors of signing DNR documents according to the logistic regression model. The unadjusted odds ratio (OR) was significant for those aged greater or equal to 85 and 75 to 84 compared to 65 to 74 (OR = 7.53, 95% CI = 2.56–22.13, and OR = 3.60, 95% CI = 1.19–10.81, respectively), admission from other departments compared to ED (OR = 2.84, 95% CI = 1.43–5.64, *p* = 0.003), higher ECOG (2–4 vs. 0–1), (OR = 2.77, 95% CI = 1.57–4.91, *p* < 0.001), lower ADL score (< 50 vs. ≥50) (OR = 4.05, 95% CI = 2.19–7.47, *p* < 0.001), higher nutrition screening score (≥2 vs. < 2) (OR = 3.70, 95% CI = 2.01–6.82, *p* < 0.001), lower serum albumin level(< 3 g/dL vs. ≥3 g/dL) (OR = 5.13, 95% CI = 2.76–9.50, *p* < 0.001), higher CCI score(> 2 vs. ≤2) (OR = 3.13, 95% CI = 1.77–5.55, *p* < 0.001), longer hospital stays (> 10 vs. ≤10) (OR = 5.46, 95% CI = 2.92–10.20, *p* < 0.001), more visits to geriatric OPD (OR = 1.07, 95% CI = 1.01–1.15, *p* = 0.025), more transfers to ICU during admission (OR = 6.11, 95% CI = 3.00–12.43, *p* < 0.001) and more vasopressor use during admission (OR = 3.62, 95% CI = 1.07–12.25, *p* = 0.029). No significant difference was noted in unadjusted OR regarding gender and number of visits to ED and admission.

**Table 3:** The predictors of DNR document using logistic regression model (n = 337)-Forward conditional method

Variables	Unadjusted OR	95% CI		*p*-value	Adjusted OR	95% CI		*p*-value
		Lower	Upper			Lower	Upper	
**Age stratification**								
75–84 v.s 65–74	3.60	1.19	10.81	0.02	2.84	0.85	9.46	0.09
≥85 v.s 65–74	7.53	2.56	22.13	< 0.001	5.94	1.78	19.76	0.004
**Gender** Male v.s Female	1.06	0.61	1.83	0.82				
**Geriatric hospitalization source**								
OPD v.s ED	1.13	0.50	2.51	0.763				
Transfer from other department v.s ED	2.84	1.43	5.64	0.003				
**ECOG** 2–4 v.s 0–1	2.77	1.57	4.91	< 0.001				
**ADL stratification** < 50 v.s ≥ 50	4.05	2.19	7.47	< 0.001				
**Nutrition Score stratification** ≥ 2 v.s < 2	3.70	2.01	6.82	< 0.001	2.71	1.33	5.51	0.006
**Albumin level stratification (g/dL)**								
< 3 v.s ≥ 3	5.13	2.76	9.50	< 0.001	3.24	1.63	6.41	0.001
Missing v.s ≥ 3	0.30	0.10	0.89	0.031	0.43	0.14	1.35	0.148
**CCI stratification** > 2 v.s ≤ 2	3.13	1.77	5.55	< 0.001	2.46	1.27	4.76	0.007
**Hospitalization day stratification** > 10 v.s ≤ 10	5.46	2.92	10.20	< 0.001				
**Geriatric OPD times in the past year**	1.07	1.01	1.15	0.02				
**Geriatric OPD stratification** Yes v.s No	1.95	1.13	3.37	0.01				
**ED or hospitalization times in the past year**	1.07	0.98	1.17	0.09				
**ICU admission** Yes v.s No	6.11	3.00	12.43	< 0.001	5.11	2.07	12.58	< 0.001
**Intubation** Yes v.s No	2.19	0.89	5.38	0.08				
**Vasopressor** Yes v.s No	3.62	1.07	12.25	0.03				

*Abbreviation*: *DNR* Do-Not-Resuscitate, *ED* Emergency Department, *OPD* Outpatient Department, *ECOG* Eastern Cooperative Oncology Group performance status, *ADL* Activity of Daily Living, *CCI* Charlson Comorbidity Index, *ICU* Intensive Care Unit

**Fig. 2:**
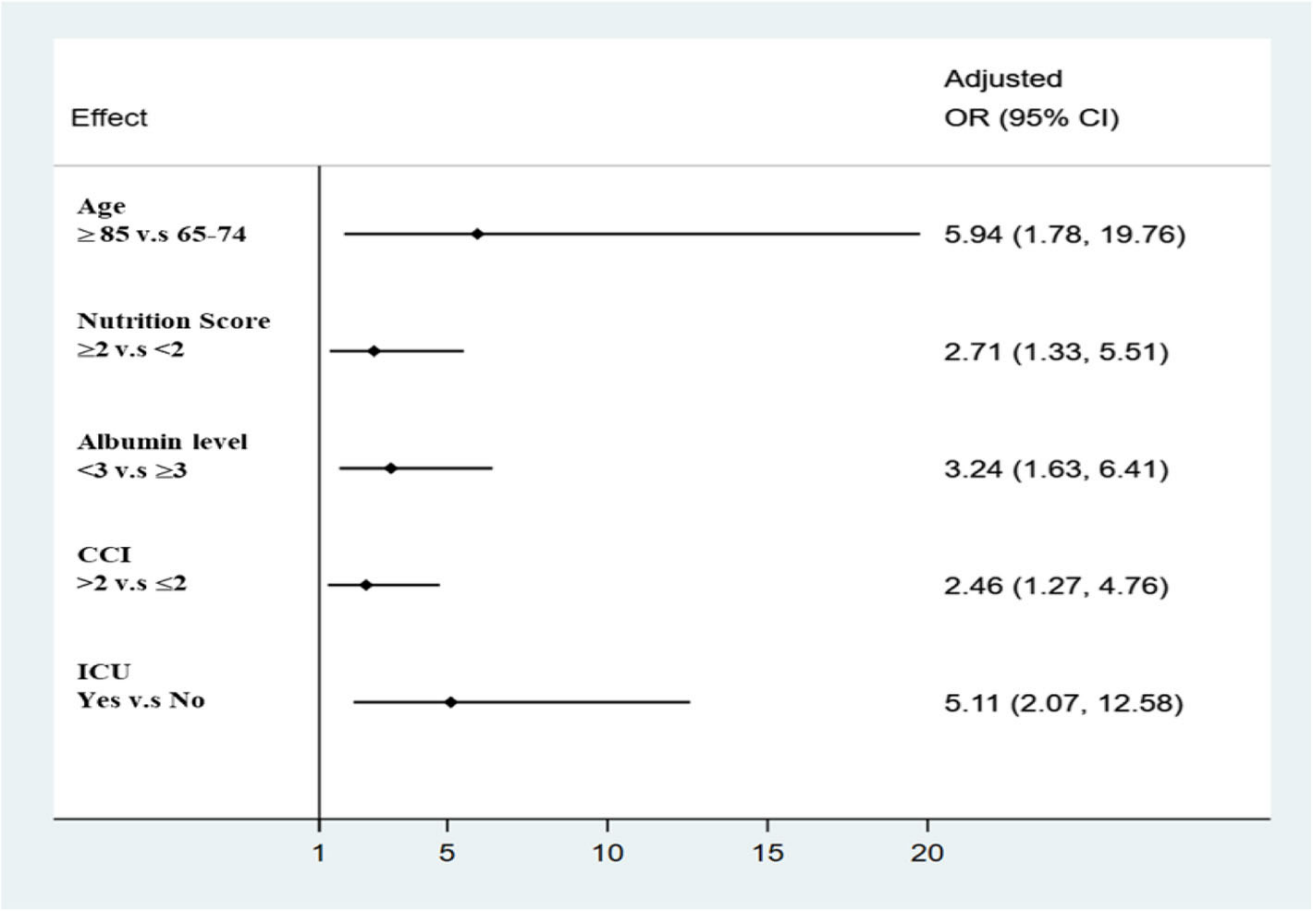
Factors associated with DNR document completion among geriatric patients. Abbreviation: OR, odds ratio, CCI Charlson Comorbidity Index, ICU Intensive Care Unit

After adjusting for other clinical factors, there were five predictors of signing DNR documents (showed in Fig. 2): 1) the adjusted OR (aOR) for age 85 or more than age 65–74 was 5.94 (95% CI = 1.78–19.76, *p* = 0.004); 2) the aOR for a nutrition screening score of 2 or more compared to less than 2 was 2.71 (95% CI = 1.33–5.51, *p* = 0.002); 3) the aOR was 3.24 in patients with serum albumin levels less than three g/dL compared to those higher than 3 g/dL (95% CI = 1.63–6.41, *p* = 0.001); 4) the aOR for CCI scoring higher than 2 compared to 2 or less was 2.46 (95% CI = 1.27–4.76, *p* = 0.007); 5) the aOR for patients transferred to ICU than not transferred was 5.11 (95% CI = 2.07–12.58, *p* < 0.001).

## Discussion

Our study found five independent factors for signing DNR documents for patients hospitalized in the geriatric ward, very-old people (age 85 or more) and transfer to ICU during hospitalization, lower albumin levels, higher nutrition screening scores, and higher comorbidities.

Similar to the present study that the oldest-old patients aged 85 or higher were more likely to have a DNR status than younger patients (age 65–74), a study in the geriatric ward of an Australian tertiary hospital found that older age was related to more acute resuscitation plan (ARP) discussions deciding to have CPR or conservative and comfortable care in the end-of-life period, with patients aged 85 to 94 having 1.65 times ARP (95% CI = 1.02–2.68), and those aged 95 or more had 9.59 times more than those less than 85 (95% CI = 2.13–43.21, *p* = 0.003) [16]. A retrospective study of ED patients with subsequent hospitalization in a university hospital found that older age was associated with do not attempt resuscitation (DNAR) documents (OR = 4.0, 95% CI = 3.6–4.3) [17]. A healthcare project in California showed at the OR for DNR status of age per 5-year increase was 1.46 (95% CI = 1.45–1.47) [18]. Also, a previous study of individuals aged 65 and older in a tertiary hospital revealed patients in the DNR group were significantly older (85.8 vs. 79.6, *p* < 0.001) [19].

According to past studies, patients with lower albumin levels are more likely to sign DNR documents. A study of patients aged 90 or more hospitalized with acute infection showed that 70 patients (43.2%) had hypoalbuminemia with a serum albumin level of less than 35 g/L and that the albumin level was independently associated with in-hospital mortality (OR: 0.86, 95% CI = 0.78–0.95, *p* = 0.004) [20]. In a study of 7279 patients by chart review, the serum albumin level was negatively associated with age and Nutritional Risk Screening Score [21]. In a Japanese study of community-acquired pneumonia older patients, serum albumin less than 2.5 g/dL was a strong factor for DNR orders (aOR 7.46, 95% CI = 1.466–37.908, *p* = 0.015) [22]. A study conducted in the ICU of a tertiary hospital of 103 patients aged ≥18 years with metastatic solid organ malignancies revealed that the lowest albumin level (HR: 1.10, 95% CI = 1.04–1.15) was strongly related to survival [23]. In our study, a serum albumin level less than 3 g/dL was also strongly associated with DNR orders. A lower albumin level might result in a poorer prognosis, so patients with lower albumin levels might be more likely to sign DNR documents.

Malnourished older patients might have a poorer prognosis. In a Korean study of very elderly (age 90 or more) patients admitted to ICU, poor nutrition was related to higher mortality (HR: 14.918, 95% CI = 2.998–74.243, *p* = 0.001) [24]. Another Korean study in a tertiary hospital with 131 older patients diagnosed with community-acquired pneumonia found that patients classified as malnourished had 2-year mortality OR of 3.06 (95% CI = 1.44–6.50, *p* = 0.004) [25]. In our hospital, the screening system with a higher score was related to longer hospital stays, lower albumin, lower hemoglobin, and total lymphocyte count [26]. Typically, geriatricians do not routinely check albumin levels, and the nutrition screening system could replace albumin to better predict older patients’ condition, as in the present study, a higher nutrition screening score indicated a poorer nutrition status. Thus, patients with a higher nutrition screen score were more likely to be assigned DNR during hospitalization.

Poor survival condition is common among geriatric patients with multiple comorbidities. Generally, clinical medical staff use CCI to evaluate the severity of patients’ comorbidities. In a study of patients hospitalized for sepsis and having a DNR order 24 h after admission, the CCI 1–3 had an OR of 1.63 (95% CI = 1.43–1.86) compared to CCI 0 (95% CI = 3.33–4.73) [27]. In the study of 10,529 older people mentioned previously, the CCI of the patients in the DNR group was 3.0 compared to 2.5 in the non-DNR group (*p* < 0.001 )[19]. In a study in a teaching hospital in Netherland, 823 patients had DNR orders, and 980 patients did not, with those with DNR orders having a mean CCI of 1.7 compared to 1.2 in the non-DNR group (*p* < 0.001) [28]. Our study found that a CCI over 2 was a significant factor in DNR assignment, therefore such a clinical finding could prompt clinical medical staff to evaluate the patient’s prognosis and discuss DNR documents earlier.

Previous literature mostly focused on DNR and mortality in ICU rather than the higher chance of transfer to ICU in the DNR group than the non-DNR group (13.3% vs. 9.4%, *p* < 0.001) [19]. In a study of 712 ICU patients, the OR of mortality in severe sepsis and septic shock patients with DNR was 6.13 (95% CI = 2.66–14.10, *p* < 0.001), [29] whereas the RR of mortality in patients with DNR was 2.39 (95% CI = 1.92–2.99) in another study [30]. The critical situation might prompt the medical team to discuss resuscitation plans for patients with poor prognoses requiring intensive medical care. In our study, transfer to ICU was a strong factor for DNR documents in geriatric ward patients, possibly because ICU admission gave the medical team opportunities to discuss ACP.

There are some limitations to our study. First, the clinical data of study subjects were from medical charts during their admission to geriatric wards between January 2018 and December 2019, the DNR status might have changed after that study period which information was lacking. This misclassification of the outcome was likely to be random and underestimated the significance of some factors. Since this is a cross-sectional study and the clinical guideline of initiating the discussion of ACP and DNR signing has not yet been established in Taiwan, this study cannot address that issue. Second, multiple admissions other than the same patient’s most present admission were excluded in the non-DNR group, and the most present admission might not be representative of the patient’s clinical condition. Finally, patients were not classified by diseases in the DNR group and the disease itself might also have influenced patients or their families to sign a DNR.

## Conclusion

In conclusion, five independent factors of DNR documents in patients hospitalized in the geriatric ward were identified. Advanced age, low albumin level, high risk of malnutrition, high comorbidities, and transfer to ICU were significantly associated with assigning a DNR. In clinical practice, the medical team should start timely ACP discussions with the patients and their families if they have any of the clinical factors mentioned above to reduce futile medical treatment and prevent patient suffering. Further prospective studies are needed to investigate the clinical factors relating to ACP discussion and DNR assignment.

## Data Availability

The datasets used and analyzed in the current study are available from the corresponding author on reasonable request.

## Abbreviations

DNR: Do-Not-Resuscitate
ACP: Advance care planning
CGA: Comprehensive geriatric assessment
CPR: Cardiopulmonary resuscitation
ADL: Activities of daily living
AD: Advance directives
POA: Power of attorney
EMR: Electronic medical records
ECOG: Eastern Cooperative Oncology Group
STROBE: Strengthening the Reporting of Observational Studies in Epidemiology
CCI: Charlson Comorbidity Index
ED: Emergency Department
OPD: Outpatient department
ICU: Intensive care unit
SGA: Subjective Global Assessment
BMI: Body mass index
BW: Body weight
TPN: Total parenteral nutrition
NPO: Nil per os
CKD: Chronic kidney disease
AAD: Against advice discharge
ID: Identity numbers
